# Spatial warping by oriented line detectors can counteract neural delays

**DOI:** 10.3389/fpsyg.2013.00794

**Published:** 2013-11-01

**Authors:** Don A. Vaughn, David M. Eagleman

**Affiliations:** ^1^Department of Neuroscience, Baylor College of MedicineHouston, TX, USA; ^2^Department of Psychiatry, Baylor College of MedicineHouston, TX, USA

**Keywords:** neural delays, neural latency, orientation tuning, prediction, postdiction, hering illusion, spatial cognition, time and motion studies

## Abstract

The slow speed of neural transmission necessitates that cortical visual information from dynamic scenes will lag reality. The “perceiving the present” (PTP) hypothesis suggests that the visual system can mitigate the effect of such delays by spatially warping scenes to look as they will in ~100 ms from now (Changizi, 2001). We here show that the Hering illusion, in which straight lines appear bowed, can be induced by a background of optic flow, consistent with the PTP hypothesis. However, importantly, the bowing direction is the same whether the flow is inward or outward. This suggests that if the warping is meant to counteract latencies, it is accomplished by a simple strategy that is insensitive to motion direction, and that works only under typical (forward-moving) circumstances. We also find that the illusion strengthens with longer pulses of optic flow, demonstrating motion integration over ~80 ms. The illusion is identical whether optic flow precedes or follows the flashing of bars, exposing the spatial warping to be equally postdictive and predictive, i.e., peri-dictive. Additionally, the illusion is diminished by cues which suggest the bars are independent of the background movement. Collectively, our findings are consistent with a role for networks of visual orientation-tuned neurons (e.g., simple cells in primary visual cortex) in spatial warping. We conclude that under the common condition of forward ego-motion, spatial warping counteracts the disadvantage of neural latencies. It is not possible to prove that this is the purpose of spatial warping, but our findings at minimum place constraints on the PTP hypothesis, demonstrating that any spatial warping for the purpose of counteracting neural delays is not a precise, on-the-fly computation, but instead a heuristic achieved by a simple mechanism that succeeds under normal circumstances.

## Introduction

It has traditionally been proposed that geometric illusions result from angle overestimation (Hering, 1861; Wundt, 1862; Holt-Hansen, 1961; Prinzmetal and Beck, 2001), presumably as a result of lateral inhibition in visual cortex (Blakemore et al., 1970) or a bias in extrapolating 3D angle information from 2D projections (Nundy et al., 2000; Howe and Purves, 2005). However, a recent framework by Changizi and colleagues suggests that several geometric illusions are caused instead by temporal delays with which the visual system must cope (Nijhawan, 1997; Changizi, 2001; Changizi and Widders, 2002). In this framework, the visual system extrapolates current information to “perceive the present” (PTP): instead of providing a conscious image of how the world was ~100 ms in the past (when signals first struck the retina), the visual system estimates how the world is likely to look in the next moment.

Despite its theoretical importance, the temporal hypothesis is supported by little direct data: it has not been unequivocally pitted against traditional frameworks, it is not known whether it would operate in a rule-based or direct manner, and there are no clues to its possible neural bases.

**Figure 1:**
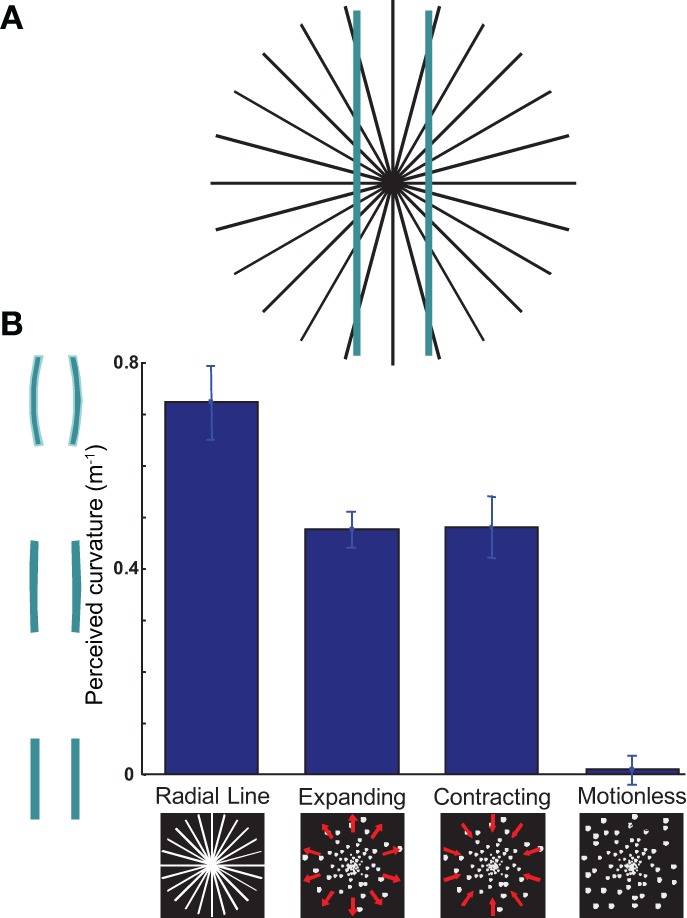
**The Hering illusion can be induced by expanding or contracting dot motion. (A)** Illustration of the Hering illusion: the straight bars appear bent. **(B)** The bars bend in the same direction whether presented against a background of radial-lines, expanding optic flow, or contracting optic flow. Against a background of motionless dots, illusory bending disappears. Ordinate values show the negative of the curvature required to nullify the illusion. *n* = 13, error bars SEM.

To test the temporal hypothesis, we capitalized on the Hering illusion (Figure 1A). The PTP hypothesis proposes that the background of radial lines simulates optic flow, causing the visual system to assume forward ego-motion and to extrapolate the appearance of the parallel bars to the next moment. Because objects closest to the horizontal plane move fastest during forward motion, this generates the illusory percept that the two parallel bars bend outward. Imagine driving on a suspension bridge toward two of its pillars: from a distance the pillars appear as parallel lines. As you approach, the pillars move farther apart at eye level, but their distant tops still appear close together.

## Methods

### Apparatus

Stimuli were displayed on a 19″ Dell monitor at a resolution of 1280 × 1024 pixels and a refresh rate of 120 Hz. Eight participants observed stimuli in a dark room, at ~0.59 m from the display.

### Participants

Thirteen subjects (5 women) participated in Experiment 1, eight (4 women) in Experiment 2, and nine (4 women) in Experiment 3. All participants were naive regarding the purpose of the experiments, had normal or corrected-to-normal vision, and signed an informed consent statement approved by the Baylor College of Medicine Institutional Review Board.

### Stimuli

On each trial, participants fixated on a red cross in the center of the screen and were presented with a background of radial lines, dots in expanding or contracting motion, or motionless dots. In all four cases, the background persisted until the participant registered an answer. The radial lines were equally spaced, subtended 17° of visual angle, and had a luminance of 11 cd/m^2^. With an average luminance of 20 cd/m^2^, each of the 600 dots subtended between 0.05 and 0.16°. The dots were displayed to imply the observer was moving forward or backward at 0.12 m/s. To achieve this, dots were randomly initialized throughout an imaginary 3D space in front of the observer. Each frame, dot positions in the imaginary depth plane were updated, and each dot was then rendered to the screen consistent in its new location, giving the dots a radial inward or outward trajectory. Consequently, dots had a larger radial displacement each frame at the outer-edges of the screen than at the focus of expansion; velocity was not constant across a dot's lifetime. Two bars, each 2° of visual angle from the vertical meridian, repeatedly flashed over the dot pattern for 80 ms with an interstimulus interval of 1 s until the participant registered an answer. The bars were generated as segments of a circle, which for each trial was randomly assigned a curvature between ±2 m^−1^ (0 is a straight vertical line). Bar length of 10.6° was held constant across all curvature values. Participants ran each condition 3 times. On each trial, the initial curvature of the two bars was randomized to one of 33 values (symmetric around 0). With the left and right arrow keys, participants adjusted the curvature until the two bars appeared subjectively straight (nullification technique).

Experiment 2 (prediction and postdiction) presented 5 durations of the background optic flow (40, 80, 160, 320, 640 ms). In prediction trials, the optic flow ended with the offset of the 80 ms bars; in postdiction trials, the background motion appeared with their onset. The interstimulus interval consisted of a 1 s blank screen, a 4 s 1/f static noise grating (uniquely generated on each trial), and another 1 s blank screen; these measures were included to prevent any motion aftereffect between presentations. Participants watched as many presentations as desired to adjust the curvature of the bars to nullify the illusion. Each condition was presented 3 times.

Using the contracting and expanding portions of the first experiment, the third experiment varied bar duration and optic flow speed. For each trial, bar duration was randomly selected to be 40, 80, 160, 320, or 640 ms, or continuously present until the participant registered an answer. Using the same method as Experiment 1, implied ego-speed was 0.12 m/s or 0.32 m/s. Participants ran 2 trials for each combination of speed, duration, and optic flow direction.

## Results

Participants viewed two bars flashed above a background of radially expanding or contracting dots (optic flow; see Methods). In randomly interleaved trials, radial lines or a control background of motionless dots were used. The bars were flashed for 80 ms with an interstimulus interval of 1 s.

Figure 1B shows the average curvature required to nullify the illusion (i.e., to make the bars appear straight). The radial line, expanding, and contracting backgrounds give rise to the Hering illusion [Figure 1B, *p* < 0.001 *t*-test; *t*_(12)_ = 10.14, 13.53, 8.19 respectively] while the motionless background does not [*p* = 0.73 *ns t*-test; *t*_(12)_ = 0.35]. Strikingly, the magnitude and direction of the illusion are nearly identical in both the expanding and contracting cases: whether the dots moved toward or away from the center, the bars appear to bow outward [paired *t*-test *ns p* = 0.93, *t*_(12)_ = 0.10; see demonstration at eaglemanlab.net/hering]. Note that the radial line condition induced the largest effect size; we suggest this would be consistent with optic flow at higher velocities becoming indistinguishable from radial lines.

At first glance, the bowing of the bars during contracting motion would seem to refute the PTP framework: an active temporal extrapolation of the scene should make the bars bend in the other direction. However, backward motion is ecologically rare, and backward extrapolation would provide little information as approaching objects would not be in the visual field (Changizi and Widders, 2002). It therefore appears plausible that a mechanism which evolved to temporally extrapolate based on optic flow might be directionally insensitive, always equating flow with forward ego-motion. Such a bias would similarly explain why observers generally perceive ambiguously forward or backward motion as forward motion (Lewis and McBeath, 2004). Thus, if the Hering illusion is caused by spatial warping to account for neural delays, we can refine our hypothesis about its mechanism and conclude that the warping operates heuristically, succeeding only in the common situation of forward motion and producing a disadvantageous percept in backward motion.

**Figure 2:**
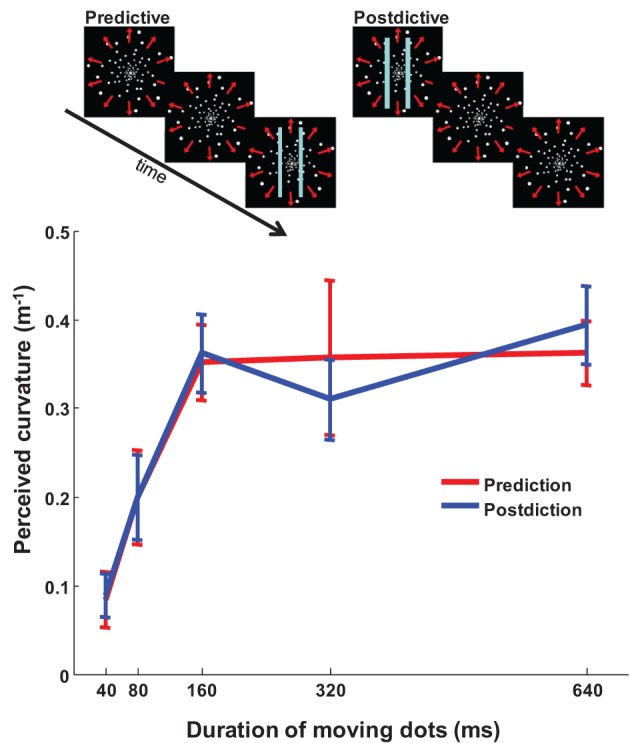
**Peri-dictive warping of the bars.** The magnitude of the illusion is identical whether background motion precedes the presentation of the bars (prediction) or follows it (postdiction). Results reveal a window of motion integration between 80 and 160 ms. In both conditions the bars flashed for 80 ms; the optic flow pattern was followed by a blank screen for 1 s, a noise grating for 4 s, and another blank screen for 1 s to eliminate motion after effect from one trial to the next. *n* = 8, error bars SEM.

We next investigated whether the putative temporal mechanisms are strictly predictive (as the PTP hypothesis posits) or might also be postdictive (Eagleman and Sejnowski, 2000). To address this, we had participants view a 1 s expanding optic flow pattern offset-aligned with 80 ms bars (predictive case) or onset-aligned (postdictive case; Figure 2). If optic flow induces spatial warping by extrapolation, any optic flow *after* the presence of the bars should have no effect on the illusion magnitude. We found, in contrast, that information collected in a ~80 ms window on either side of the bars contributes equally to the spatial warping [Figure 2; Two-Way ANOVA, motion duration *p* < 0.001, *F*_(4, 74)_ = 73.56; pre/postdiction *ns p* = 1.00, *F*_(1, 74)_ = 0.00]. In other words, the effect is not merely postdictive or predictive, but symmetrically peri-dictive: there is a symmetrical temporal window of motion integration around the flashing of the bars.

**Figure 3:**
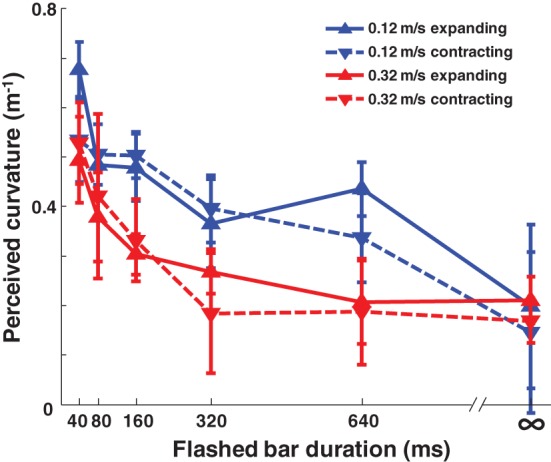
**The magnitude of the Hering illusion decreases with increasing bar duration and dot speed, both of which give evidence that the bars should not be expected to move with the background.** Accordingly, the warping of the bars diminishes. *n* = 9, error bars SEM.

Having established that implied motion evokes this illusion, we next investigated the effect of modulating the two main temporal parameters: background dot speed and the duration of the bars' presence. Participants viewed the expanding and contracting conditions of the experiment at two different background speeds with five different bar durations. The magnitude of the illusion was significantly reduced by increased bar duration [Figure 3; *p* < 0.001, *F*_(5, 208)_ = 14.52] and by increased background speed [*p* < 0.001, *F*_(1, 208)_ = 9.09, Three-Way ANOVA].

These results do not seem consistent with the angle overestimation hypothesis (AOH; Prinzmetal and Beck, 2001), as the AOH might have predicted that a longer bar duration would give a clearer signal of the intersection angle, making the effect larger. However, we find the opposite: longer bar durations decrease the effect magnitude. Moreover, the background dots increasingly look like lines as their speed increases, which would again make the intersection angle clearer, predicting a larger effect at faster speeds if the AOH were true; we find instead, a decreased effect with increased dot speed. We note, however, that the results could be consistent with the AOH if the visual system instead treats increased dot speed and decreased bar duration as low-contrast signals, given that contrast does effect the Hering illusion's magnitude (Astor-Stetson and Purnell, 1990).

Instead, we suggest that a continued presence of the bars evinces that the bars are not moving relative to the observer even while the dot pattern is moving, allowing the visual system to reduce the coupling between the bars and the background, and therefore to warp them less. Such a variable coupling can further explain why increased dot speed decreases the illusion magnitude: at faster speeds, the bars should change location even more if they were part of the background. Thus, an increased passage of optic flow for a fixed duration serves as mounting evidence that the bars are separate from the background.

Although a different geometric illusion against a background of expanding dots had previously been demonstrated (Changizi et al., 2008), the importance of the present findings lies in the equivalence of the illusion in both forward and backward motion, both predictively and postdictively, and as a function of the degree to which the bars are expected to change. First, these findings indicate that the spatial warping is a heuristic rather than an on-line computation. Second, if we had merely shown the illusion with expanding motion, our findings could have potentially been explained by perceptual displacement of the lines by the background motion (Ramachandran and Cavanagh, 1987; Festa-Martino and Welch, 2001; Eagleman and Sejnowski, 2007); the illusion with contracting dots rules out motion capture as a possible explanation for this phenomenon (Figure 1).

Third, our demonstration that the Hering illusion is symmetrically induced by expanding or contracting optic flow either preceding or following the presentation of the bars unmasks clues about underlying neural mechanisms. Specifically, parsimony might suggest a single neural mechanism with two properties: (1) it is equally sensitive to static lines and antiparallel motion and (2) has an 80 ms symmetrical temporal integration window. Neurons in area MT do not meet the criteria: they are typically responsive to movement in a particular direction, and either do not respond or sometimes show suppressive effects to the opposite direction (Snowden et al., 1991; Bradley et al., 1995). Similarly, many neurons in area MSTd are responsive to either expanding or contracting optic flow patterns, but not both (Saito et al., 1986; Tanaka et al., 1989). Further, as a population, MSTd neurons are not responsive to radial lines. It therefore appears unlikely that the neural mechanisms of the illusion involve higher level, motion-sensitive areas like MT and MSTd. Instead, a stronger model would implicate orientation selective neurons in primary visual cortex, V1. These simple cells are sensitive to lines (Hubel and Wiesel, 1959) as well as motion streaks from dots moving at sufficient speed in either direction parallel to the preferred orientation (Geisler, 1999), and they have a temporal integration window consistent with our results. Future experiments in primates could elucidate if high-level warping of a visual scene to account for neural delays is rooted in the directionally-insensitive response of V1 neurons.

In summary, our findings indicate that the spatial warping caused by motion streaks reduces to the PTP model under the typical circumstances of forward ego-motion. This does not prove that the PTP hypothesis is the reason for the warping, but it is consistent with the possibility. Our current findings place constraints on the PTP hypothesis, demonstrating that any spatial warping for the purpose of counteracting neural delays is not a “smart,” active neural process, but instead a heuristic subserved by a simple mechanism that succeeds only under forward-moving circumstances.

## Author contributions

Don A. Vaughn and David M. Eagleman jointly designed and conducted the experiments and jointly wrote the manuscript.

### Conflict of interest statement

The authors declare that the research was conducted in the absence of any commercial or financial relationships that could be construed as a potential conflict of interest.
